# Valuing Blue Carbon: Carbon Sequestration Benefits Provided by the Marine Protected Areas in Colombia

**DOI:** 10.1371/journal.pone.0126627

**Published:** 2015-05-27

**Authors:** Tatiana G. Zarate-Barrera, Jorge H. Maldonado

**Affiliations:** Department of Economics—CEDE, Universidad de los Andes, Bogotá, Colombia; University of Auckland, NEW ZEALAND

## Abstract

Marine protected areas are aimed to protect and conserve key ecosystems for the provision of a number of ecosystem services that are the basis for numerous economic activities. Among the several services that these areas provide, the capacity of sequestering (capturing and storing) organic carbon is a regulating service, provided mainly by mangroves and seagrasses, that gains importance as alternatives for mitigating global warming become a priority in the international agenda. The objective of this study is to value the services associated with the capture and storage of oceanic carbon, known as Blue Carbon, provided by a new network of marine protected areas in Colombia. We approach the monetary value associated to these services through the simulation of a hypothetical market for oceanic carbon. To do that, we construct a benefit function that considers the capacity of mangroves and seagrasses for capturing and storing blue carbon, and simulate scenarios for the variation of key variables such as the market carbon price, the discount rate, the natural rate of loss of the ecosystems, and the expectations about the post-Kyoto negotiations. The results indicate that the expected benefits associated to carbon capture and storage provided by these ecosystems are substantial but highly dependent on the expectations in terms of the negotiations surrounding the extension of the Kyoto Protocol and the dynamics of the carbon credit’s demand and supply. We also find that the natural loss rate of these ecosystems does not seem to have a significant effect on the annual value of the benefits. This approach constitutes one of the first attempts to value blue carbon as one of the services provided by conservation.

## Introduction

Human beings depend on the oceans, coasts and seas to have access to food, energy, regulating climate, transport and recreation [1]. In tropical countries, it is estimated that more than two billion people depend on the goods and services provided by marine and coastal ecosystems [2].

Despite the variety of goods and services offered by the marine and coastal ecosystems, the fragility of the dynamics established among species and the combination of natural and human processes (intentional and non-intentional) have led to a deterioration of the ecosystems over the past few decades, putting at risk the effective and sustainable provisioning of the environmental services provided by these ecosystems. In this respect, the protection of marine and coastal areas has become a mechanism for the conservation of the affluence of goods and services that they offer to society [3].

**Fig 1:**
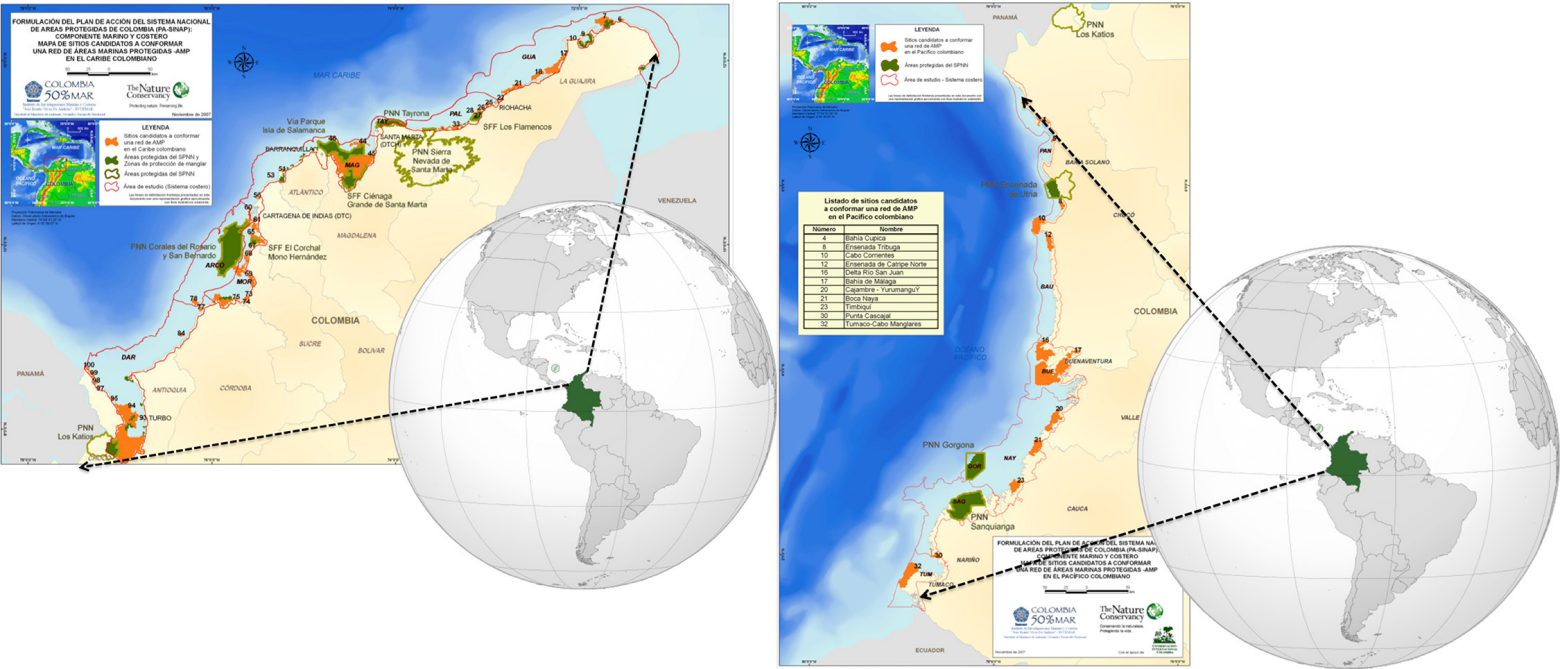
Map of Colombian current and proposed marine protected areas. (a) Caribbean Sea. (b) Pacific Ocean. Source: INVEMAR [32]

Furthermore, most countries have resorted to the establishment of marine protected areas (MPAs) as a strategy for the protection of marine biodiversity against the pressures that have affected marine health over the past few decades. This process has been accelerated by the commitment of several countries being signatories to the Convention on Biological Diversity -2004- [4], leading to an increase in the data on marine and coastal areas under protection. Colombia has also used the establishment of marine protected areas as a tool for the conservation of marine and coastal ecosystems. At present, the country has 15 national marine protected areas and a number of other regional ones, and authorities and research institutions are advancing in the design and consolidation of a subsystem (a network) of marine protected areas. With the proposed MPA subsystem (Fig 1), the total marine protected area will reach around 4 million hectares, an area equivalent to near 4% of terrestrial surface.

Despite these efforts, developing countries such as Colombia keep struggling against the lack of funding for increasing their protected areas and consolidating them as a network, in order to increase efficiency and efficacy in conservation and to fulfill the commitments required by the Convention.

The economic valuation of the services provided by the ecosystems has become a main topic with which to highlight the importance of key ecosystems for conservation and the need of funding the establishment and expansion of marine protected areas. The International Union for Conservation of Nature (IUCN), The Nature Conservancy and the World Bank recognize the relevance of assigning a monetary value to the ecosystem services in order to help emphasize how important these services are for humankind [5].

Most of the economic exercises valuing marine ecosystems have concentrated on services such as recreation, tourism and fisheries [6]. However, very few studies have dealt with valuing regulating services that provide indirect-use value such as the carbon-sequestration service provided by the marine and coastal ecosystems, known as Blue Carbon in the more recent literature [7].

The literature is as yet incipient in terms of oceanic-carbon capture and storage and the majority of studies have concentrated on detailed descriptions of its advantages over forest carbon and on the importance of their inclusion in agreements such as the Kyoto Protocol [8]. Only a few studies have concentrated on offering quantitative results in economic terms of its mitigation potential of Greenhouse Gases [9, 10]. One study, Green Payments for Blue Carbon Economic Incentives for Protecting Threatened Coastal Habitats [10] makes an approximation of the economic valuation and the establishment of monetary values for this service, focusing on emissions markets, also known as carbon markets. These emissions markets, together with the Clean Development Mechanism (CDM) and Joint Implementation (JI), are part of the strategies created at the Kyoto Protocol, and coordinated by the UN Framework Convention on Climate Change (FCCC) to reduce emissions of Greenhouse Gases (GHG) and promote initiatives that encourage adaptation to the effects of climate change.

The ecosystem service of carbon sequestration, understood as the process of capture and long-term storage of atmospheric carbon dioxide has been recognized for its contribution to climate change mitigation [11, 12]. Thus, research on its biological dynamics and the development of mechanisms such as emissions markets have raised awareness and have generated incentives for the conservation of ecosystems that provide this service. However, the recognition of marine ecosystems within the dynamics of global climate is still incipient, hence quantifying benefits is necessary in order to highlight the value associated to the service that those ecosystems provide to society.

In this context, the objective of this study is to determine the economic value associated to the services of oceanic-carbon capture and storage provided by the network of marine protected areas. This scenario is applied to conditions in Colombia, a country engaged in the consolidation of a subsystem of marine protected areas through the increase of the zones under protection [13]. To achieve this objective we propose an economic model to quantify the benefits associated to the oceanic carbon sequestration service provided by a set of MPAs, simulating a hypothetical market for oceanic carbon. In this model, we construct a benefit function that considers items such as carbon capture rates, seabeds and biomass storage, and ecosystems’ annual loss rates, as well as different discount rates and the hypothetical market and price simulations as tools to monetize these services.

The valuation of the services provided by marine ecosystems such as carbon capture and storage seeks to associate an economic value to such services to generate economic incentives that support their conservation. The protection of new hectares of marine ecosystems, including those that capture carbon, will be directly visible in the total storage and in the annual capture rates that are considered stable because they are under a protection scheme. This implies that the protection scheme will guarantee that the ecosystems are free from human imposed threats such as habitat transformation, so that the dynamics associated to their health are appropriate and make it possible to guarantee that their processes of capture and storage are kept. It is noteworthy that the protection does not mean that the ecosystems will not be vulnerable to exogenous phenomena such as climate change and natural degradation processes. Similarly, we cannot affirm that the lack of a protection scheme will imply that the provision of these services falls to zero. Hence, even though there is room for a potentially limited additionality, we are assuming that in a carbon market, the effort of protection would be recognized as a sufficient condition for recognizing its value.

This document is structured as follows: following this introduction, we present a literature review of topics pertaining to the study, as well as the proposed methodologies for the valuation of the environmental service being studied. Next, we present the results for the valuation of blue carbon including three different scenarios and some sensitivity analysis. Finally in the last section, we present a discussion of the results obtained.

## Background

**Fig 2:**
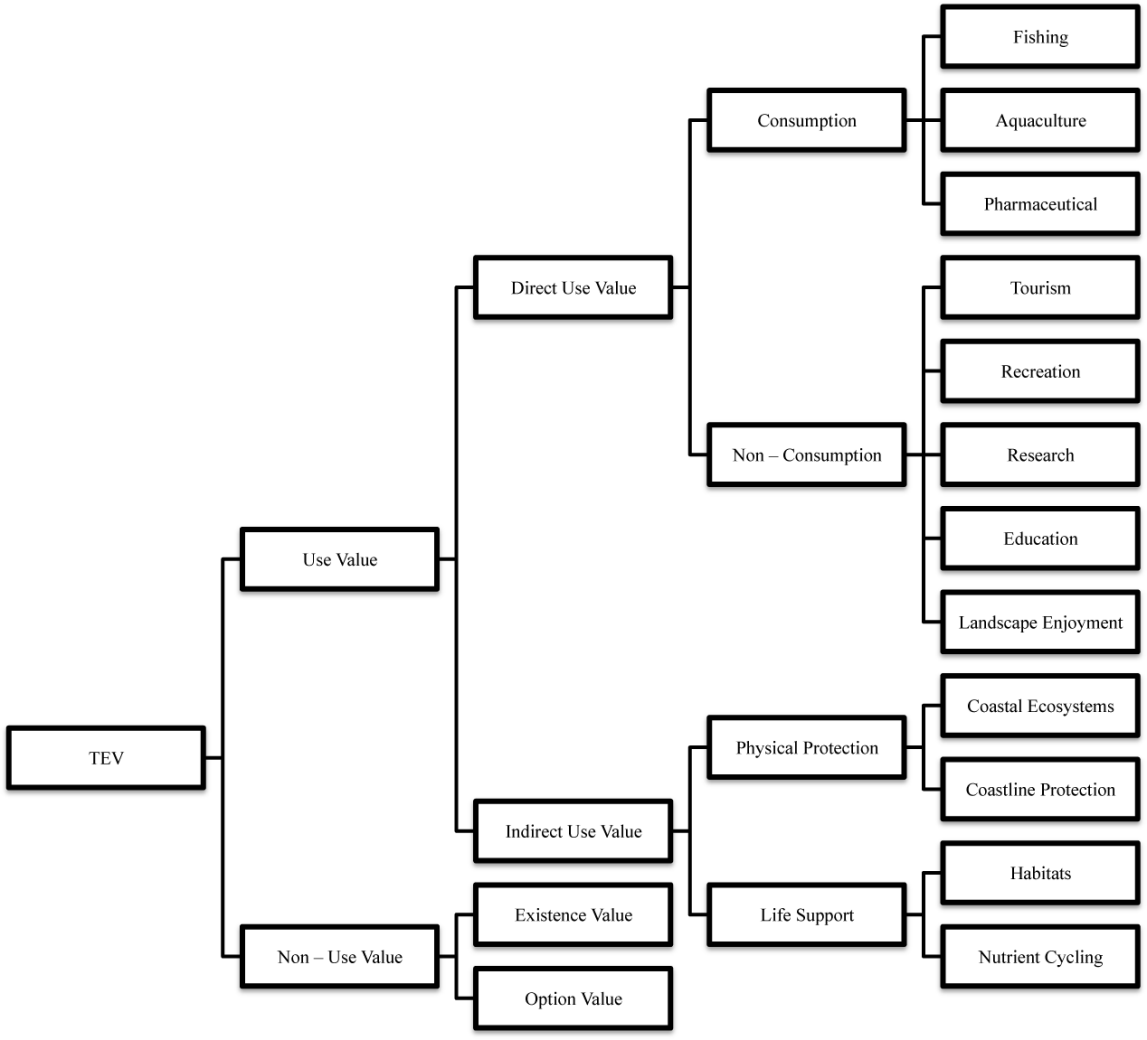
Total Economic Value (TEV) and its components applied to marine and coastal ecosystems. Source: adjusted from Boyle & Bishop [15] and Emerton [16].

In 2005, the Millennium Ecosystem Assessment (MEA) defined ecosystem services as the benefits that human beings obtain from ecosystems. These services include provisioning services such as food, water, timber products, fibers and genetic resources; regulating services such as coastal protection from storms, floods and waves, erosion prevention, climate regulation, waste treatment, water quality, and carbon sequestration (capture and storage); cultural services such as recreation, landscape enjoyment and spiritual wellbeing; and life supporting services such as seabed formation and nutrient cycles [14]. It has been largely discussed that granting a monetary or economic value to the services that an ecosystem provides can help to prove the importance of its survival [5]. The economic value of an ecosystem or its services must consider the concept of Total Economic Value (TEV), which comprises the use and non-use values (see Fig 2), the former being made up of the direct and indirect use values and the latter being associated to the existence and option values [15, 16]. Valuation exercises based on revealed-preference methods concentrate on estimating direct-use values, because methods based on stated preferences fit better in valuing non-use value (option and existence). But traditionally, the valuation of regulation services, which generate indirect-use values, is less frequent in the literature.

The estimation of indirect-use values associated to some of the services that are not directly observable, such as carbon capture and storage, can provide valuable information about the importance of related ecosystems and bring incentives for and justify the policies that support marine protected areas [17].

One of the key indirect services provided by the marine and coastal ecosystems is the service associated to carbon sequestration, which includes both capture and long-term storage [18]. From all the biological carbon (Green Carbon) captured in the world, more than half (55%) is captured by marine organisms (Blue Carbon), which means that the oceans play a significant role in the global carbon cycle, not only because they represent the largest carbon reserves but also because they can store and redistribute it through the cycle [19]. In the same study, Nellemann *et al*. affirm that approximately 93% of the Earth’s carbon dioxide is stored and undergoes its cycle in the oceans. Similarly, the coastal ecosystems are recognized as the largest carbon sinks, given that they store large quantities of carbon both in their vegetation and the seabed [20]. In fact, the oceans’ plant ecosystems, in particular mangroves and seagrasses—despite covering only a relatively small percentage of the surface—constitute reserves of more than 50% of the organic carbon stored in marine sediments and therefore, are the most intense reserves on the planet [19].

Mangroves fix and store significant quantities of carbon and play an important role in the carbon sequestration process [21]. It is estimated that these ecosystems absorb around 25.5 million tons of carbon per year [22]. Although mangroves play an important role in the global-carbon cycle, the loss of 35% of mangrove ecosystems around the world over the past two decades has led to the emission of large quantities of stored carbon and therefore, contributed to global warming [23].

In contrast, coral reefs contribute with between 7 and 15 percent of the global production of calcium carbonate, contributing to carbon sequestration [24]. In this way, the sedimentary carbonates, including corals, coralline algae and the shells of other marine organisms, are positioned as the planet’s largest carbon reserves, and fluctuations in these reserves influence the concentration of carbon dioxide in the atmosphere. However, the chemistry of the system is such that even though the oceans are carbon sinks (absorbing carbon dioxide), coral reefs are net sources or producers of carbon dioxide, even if only on a small scale, through the calcification process [24].

With respect to blue carbon, Sifleet et al. [20] synthesize the results of the main biological censuses associated to the measurement of characteristics such as specific capture and storage rates for each relevant ecosystem. These authors also affirm that in the same way that carbon storage has to be considered in the decisions on how to manage the habitats, it is also necessary for the land-owners and coastline administrators to not transform the ecosystems and the environmental services that they provide, in particular that of carbon capture and storage [21]. Given that markets do not easily capture the value that ecosystem services provide, these values are not considered by the policymakers, leading to the excessive destruction of habitats [10]. This is of concern because the transformation of coastal ecosystems today, with loss rates between 2 and 15 times greater than tropical forests [19], generates significant GHG emissions. In fact, loss by conversion from marshes, mangroves and seagrasses can imply a release of 0.15–1.02 billion tons of carbon dioxide, equivalent to 3–19% of emissions from deforestation globally [8].

The global efforts made to reduce greenhouse gas emissions have led to the creation of the emissions trading system, known as the Carbon Market, which generates economic incentives to encourage the land-owners not to transform the forest ecosystems. However, the inclusion of marine ecosystems in the carbon market is still being negotiated because the scientific understanding and consensus about the blue carbon mitigation potential have not been sufficiently developed until now [25], while there are other initiatives that are being studied at the international level such as Reducing Emissions from Deforestation and Forest Degradation (REDD+).

Murray et al. [10] present an economic model that allows an approximation to the valuation of Blue Carbon in a hypothetical carbon market in which marine ecosystems are included. Through case studies, they find, for example, that for mangroves in the tropical parts of Asia, the market value of carbon which is not released into the atmosphere may be between USD$5,000 and $37,000 per hectare, in scenarios whereby the price of carbon is between USD$5.00 and USD$30.00/tCO_2_e [10]. Loss of marshes, mangroves and seagrasses might imply economic costs of USD$6–42 billion annually [9].

Considering the above, in the more recent literature there have been different approaches regarding the determination of the carbon price to be used. The carbon price should reflect the marginal cost of issuing an additional unit of Greenhouse Gas, as an organization or country wishing to reduce emissions may only make it to the point where the marginal cost of reducing an additional unit begins to be greater than the price paid [26]. However, the problem with the determination of this rate is how to implement it, since it is difficult to accurately incorporate climate variables and impacts in the estimations, as well as there being an ample set of opportunities to reduce these emissions. In theory, it should be equal to the present value of the economic damage generated by an additional unit of emitted GHG, in trying to reflect what a society would be willing to pay today in order to prevent future damage from GHG emissions, commonly termed Social Cost of Carbon (SCC) [27].

The Intergovernmental Panel on Climate Change studied the benefits associated to GHG mitigation, and through an “integrated assessment model” found that the social cost of carbon emissions in 2008 was between USD$12.00 and 17.00/MgCO2e [Mega gram of Carbon Dioxide-Equivalent] [28]; however, as the Environmental Protection Agency (EPA) observed, the IPCC ensures that the concept associated to SCC underestimates the environmental damage caused by GHG emissions, because the integrated assessment models which are used to estimate the SCC do not include important impacts of climate change [29].

As the purpose of this manuscript is to quantify the contribution associated to oceanic carbon sequestration provided by the Subsystem of Marine Protected Areas for Colombia (SMPA), the strategy consists of designing an economic model that allows to describe and quantify the benefits that the country will obtain within the framework of a hypothetical carbon market such as the one set out by Murray et al. [9].

## Methodology

In this section we show how to construct the proposed hypothetical emissions market. First, we present the model that describes the benefits associated with the service of carbon capture and storage. Then, we describe the details about the data sources used for the simulation, which include the major characteristics of ecosystems that provide this service, the discount rates to be chosen, and the approach for estimating the future price in a specific emissions market.

### Carbon Sequestration Model

Carbon capture and storage involve three components [10]: The first component refers to the annual rates of carbon capture per surface unit of each ecosystem *i* (*S*_*i*_), defined as the annual flow of organic material transferred to a hectare of seabed. The second component refers to the quantity of carbon stored as biomass that can be denoted as stock and is associated to the carbon stored in leaves and roots as a product of a previous capture. The third component refers to the stock of carbon stored in the organic seabed located below the coastal ecosystems [10]. The latter two components are defined as *EC*02_*i*_, referring to the emissions avoided through not transforming each ecosystem, related to the carbon stored as biomass and in the seabed.

Let the marine area currently under protection be denoted by *A*; and let the new marine area to be protected be denoted by *R*. After increasing the network of marine protected areas, the total area under protection would be *A* + *R*. Now, *q*_*im*_ is defined as the area occupied by each ecosystem *i* in an area *m* that captures carbon (*m = A* or *R*); in the case of the Colombian Caribbean Sea, the relevant ecosystems are mangroves and seagrasses, and in the case of the Colombian Pacific Ocean only mangroves, as there are no seagrasses present in this region.

Considering the above it is possible to build an expression that describes the carbon capture rate in each period *t* as:

$$C_{m}=\overset{n}{\underset{i=1}{\sum }}q_{im}S_{i}\left(1-x_{i}\right)$$ (1)

Eq (1) represents the rate of capture of any area in each period, where *n* refers to the number of ecosystems that capture carbon. This expression indicates that the Megagrams of carbon dioxide equivalents (MgCO2e) captured will depend on the specific rate of capture of each ecosystem (*S*_*i*_), the hectares that this ecosystem occupy in the area (*q*_*im*_), and the annual rate of loss (*x*_*i*_) of ecosystems that sequester carbon. The inclusion of an annual rate of loss *x*_*i*_ under the existence of protection figures can be explained as being due to uncontrollable phenomena such as natural degradation rates pertaining to each ecosystem and to the effects of climate change. In this way, we could consider degradation rate *x*_*i*_ as a function of the natural rate of degradation specific to each ecosystem, as well as parameters such as temperature, sea level, precipitation and other elements that have suffered modification as a consequence of climate change. The *x*_*i*_ parameter will allow building different scenarios to analyze the effect of the aforementioned capture rates and quantities of stored carbon.

The expression that describes carbon storage can be constructed in a similar way to the annual capture rate, including both the carbon stored as biomass, and the carbon stored in the seabed, through the variable *EE*_*i*_:

$$Q_{m}=\overset{n}{\underset{i=1}{\sum }}q_{im}EE_{i}\left(1-x_{i}\right)$$ (2)

Eq (2) defines the total carbon storage in biomass and seabed of any area *m*, where *EE*_*i*_ is defined as the amount of carbon stored per hectare in the seabed *g*_*i*_ and in the vegetation *v*_*i*_ of the ecosystem, i.e. *EE*_*i*_ = *g*_*i*_ + *v*_*i*_.

Now, in order to approach an economic value of the carbon sequestration service, we set out a model of benefits for any protected area *m*. To calculate these benefits, the variable *EE*_*i*_ must be modified to include the structure that guides the emissions markets and the conditions necessary for a project to be deserving emissions rights.

A project that transforms the land cover would generate two effects: first, the ability of ecosystems to capture carbon would be lost, and second the project can cause the emission of carbon dioxide into the atmosphere when carbon that is stored in the seabed is exposed to oxygen and is converted to CO_2_. In this context, following Pendleton et al. [9] and considering conservative scenarios, it is only assumed in the risk of emission of the carbon stored in the first meter depth of the seabed, as it can be potentially released into the atmosphere. Therefore, for the model we must consider only the carbon stored in the first meter of the marine seabed, and name it $EE_{i}^{'}$, which includes the carbon stored as biomass and as seabed. Let *Lm* be the carbon stored in the first meter depth of the seabed:

$$L_{m}=\overset{n}{\underset{i=1}{\sum }}q_{im}E_{i}^{'}\left(1-x_{i}\right)$$ (3)

The benefits associated to the service of capturing and storing carbon, framed into a hypothetical project that would grant Certified Emission Reduction to the country, would be the result of adding the annual flows that generate oceanic carbon capture and the amount that would be received at the end of the project period. Further, this would involve recognizing the land use protection of the ecosystem, which would avoid the emission of CO_2_ over the life of the project. Thus, the benefits for any period t during first *T* – 1 periods would be equivalent to:

$$\pi_{m}^{T-1}=C_{m}P$$

And for the last period, *T*:

$$\pi_{m}^{T}=\left(C_{m}+L_{m}\right)P$$

Where *P* refers to the expected price in the hypothetical market and *L*_*m*_ refers to emissions at risk or potentially releasable due to a project that will transform the ecosystem seabed.

Considering the above, for *T* periods we propose an expression to analyze the contribution of any area *m* within the context of a hypothetical market in order to associate a monetary value to the environmental benefit provided by marine ecosystems, due to carbon sequestration. In this context, we want to value the new MPAs in the subsystem (*R*), hence we replace the sub-index *m* which is generic for any area for the sub-index *R* for the new MPAs:

$$\Pi_{R}=\overset{T}{\underset{t=0}{\sum }}\frac{C_{R}P}{{\left(1+r\right)}^{t}}+\frac{L_{R}P}{{\left(1+r\right)}^{T}}$$ (4)

Eq (4) describes the benefits of carbon capture (*C*_*R*_) and storage (*L*_*R*_) (potentially releasable) in the MPAs subsystem *R*. Here *r* is defined as the discount rate that brings to the present value the carbon flows within a project that prevents the conversion of ecosystems.

Now, the benefits function should consider the uncertainty associated to the prices in a cap and trade market. In order to do that, we introduce a parameter *α* that captures a state of the nature that defines a given price, and includes aspects such as the uncertainty related to the post-Kyoto negotiations, the expectations associated to the completion of the first period of the Protocol and Phase II of the European Union Emissions Trading System (EU ETS), the dynamics between supply and demand that this market permits, among others. Thus, this parameter is defined as the probability that the prices are low (*P*_*L*_), and (1–*α*) the probability that the prices are high (*P*_*H*_).

Given this new parameter, the benefits function now becomes probabilistic, that is, an expected benefits function that takes the following form:

$$E\left(\pi_{R}\right)=\alpha \left(\overset{T}{\underset{t=0}{\sum }}\frac{C_{R}P_{L}}{{\left(1+r\right)}^{t}}+\frac{L_{R}P_{L}}{{\left(1+r\right)}^{T}}\right)+\left(1-\alpha \right)\left(\overset{T}{\underset{t=0}{\sum }}\frac{C_{R}P_{H}}{{\left(1+r\right)}^{t}}+\frac{L_{R}P_{H}}{{\left(1+r\right)}^{T}}\right)$$ (5)

Here we propose three possible values for *α* considering non optimistic, optimistic and neutral results for post-Kyoto negotiations, that is: (i) some countries withdraw from the Protocol, (ii) the Protocol is ratified and (iii) the future of the Protocol is as yet undefined. Therefore, for the first response, we took into account the negative effect of countries withdrawing for which *α* was arbitrarily assigned a value of 0.7; for the second response *α* was assigned a value of 0.3; and for the third response, the *α* was assigned a value equivalent to 0.5. In order to understand better the effect of changes in the parameters of interest, we calculate the elasticity of the expected benefits to changes in the parameter *α* and the elasticity to changes in the loss rate *x*_*i*_, as follows: In order to understand better the effect of changes in the parameters of interest, we calculate the elasticity of the expected benefits to changes in the parameter *α* and the elasticity to changes in the loss rate *x*_*i*_, as follows:

$$E\left(\pi_{R},\alpha \right)=\frac{\partial \pi_{R}}{\partial \alpha }\frac{\alpha }{\pi_{R}}=\frac{\alpha \left(P_{L}-P_{H}\right)}{\pi_{R}}\left(C_{R}\left(\overset{T}{\underset{t=0}{\sum }}\frac{1}{{\left(1+r\right)}^{t}}\right)+L_{R}\left(\frac{1}{{\left(1+r\right)}^{T}}\right)\right)$$ (6)

$$E\left(\pi_{R},x_{i}\right)=\frac{\partial \pi_{R}}{\partial x_{i}}\frac{x_{i}}{\pi_{R}}=\left[\left(\overset{n}{\underset{i=1}{\sum }}-q_{iR}S_{i}\right)\left(\overset{T}{\underset{t=0}{\sum }}\frac{\alpha P_{L}+\left(1-\alpha \right)P_{H}}{{\left(1+r\right)}^{t}}\right)+\left(\overset{n}{\underset{i=1}{\sum }}-q_{iR}EE_{i}^{'}\right)\left(\frac{\alpha P_{L}+\left(1-\alpha \right)P_{H}}{{\left(1+r\right)}^{T}}\right)\right]\frac{x_{i}}{\pi_{R}}$$ (7)

The signs of the elasticities are as expected. In (6) the response of the expected benefits regarding an increase of *α* is consistent with an expected reduction of the benefits, therefore the elasticity is negative. In fact, note that with an increase in *α*, the benefits diminish in proportion to the expected difference of prices (*P*_*H*_–*P*_*L*_), since by construction *P*_*L*_<*P*_*H*_.

### Data for the Model

#### Marine and coastal carbon sequestering ecosystems

The role of forest ecosystems as carbon sequestering systems is well known; however, the role of some marine and coastal ecosystems, mangroves and seagrasses as source and sink of greenhouse gases, has not been extensively studied [8]. Ecosystems such as mangroves and seagrass remove GHG from the atmosphere through the process of photosynthesis. These ecosystems sequester significant amounts of greenhouse gases, mainly CO2, and store it as biomass and in the seabeds that underlie them [10, 11, 12, 30].

In Colombia seagrasses are distributed throughout the Caribbean with an area of 43,223 ha, but there are no records of any kind in the Pacific coast [31]. Mangrove forests cover an area of almost 380 thousand hectares, 292,724 of which belong to the Pacific coast [31]. The areas of mangroves and seagrasses currently protected are approximately 93,939 ha and 7,323 ha, respectively, while for the new MPAs they will cover around 18,864 ha of seagrasses and 45,805 ha of mangroves (Table 1), 44% and 12% of total area of these ecosystems in the country, respectively [32].

**Table 1:** Distribution of Mangroves and Seagrasses in Colombia (Hectares).

	Mangroves	Seagrasses
**Caribbean**	87,230	43,223
**Pacific**	292,724	0
**Total**	379,954	43,223
**Current Protection**	93,939 *(25%)*	7,323 *(17%)*
**Subsystem Protection**	45,805 *(12%)*	18,864 *(44%)*
**Total Protection**	139,744 *(37%)*	26,187 *(61%)*

Source: Authors’ own based on[32] and [31].

Measurements of mangrove and seagrass capture and storage (in biomass and seabed) demonstrated ample variation [8, 9] [16] [20], as shown in Table 2. For this study we use the average values reported from these studies for capture, storage and potentially releasable carbon.

**Table 2:** Carbon Capture and Storage Rates for Seabed and Vegetation of Mangroves and Seagrasses.

	Mangroves			Seagrasses		
	Min	Max	Avg	Min	Max	Avg
**Capture rate (MgCO**_**2**_ **e/ha/yr)**	0.13	24	6	0	85	4
**Storage in biomass (MgCO**_**2**_ **e/ha)**	26	2,554	718	0.01	23	2.5
**Storage in seabed (MgCO**_**2**_ **e/ha)**	2,126	2,603	2,461	66	1,467	766
**Potentially releasable (MgCO**_**2**_ **e/ha)**	1,492			522		

Source: Authors’ own based on [9], [10] and [20]

Finally, the annual loss *x*_*i*_ of these ecosystems was approached according to the data provided by the Food and Agriculture Organization (FAO) for mangroves in Colombia [33]. Thus, the rates used per annum were 0.58% and 1.12%, and were the same for both mangroves and seagrasses. Given there is lack of information about the dynamics of these loss rates, we assume them to be constant over the period of analysis.

Discount Rate. An important aspect for the proposed valuation is the choice of the appropriate discount rate. Some authors suggest that the appropriate social discount rate for Colombia is 4.2% annually [34]. In contrast, the World Bank advises including rates close to 12% for developing countries like Colombia, while Harrison [35] ensures that low interest rates should be used in environmental assessments, especially when extended periods of time are considered. Given this wide set of options, choosing a discount rate for the analysis is not an easy task. Considering the sensitivity of results to the discount rate, our approach consists of three possible values to discount the stream of social benefits obtained by the capture service and oceanic carbon storage, that is: a low rate (4%) following [35], an intermediate value (6%) and a “high” value as recommended by the World Bank (9%) but not too high (i.e., 12%) because it is an environmental project.

#### Carbon Markets

The UN Framework Convention on Climate Change (UNFCCC) is a global agreement to face the impacts of climate change, which includes within its associated elements strategies to mitigate GHG emissions, initiatives for adaptation to climate change, financing mechanisms and incentives for the use of clean technologies, inter alia. UNFCCC seeks to stabilize GHG emissions in deadlines established, such that “a level that would prevent dangerous anthropogenic interference with the climate system” ensuring the aim of [36] ecosystems to adapt naturally to climate change is reached, whereby food production is not threatened and economic development proceeds in a sustainable manner.

As part of the Kyoto Protocol the Clean Development Mechanism (CDM) was agreed which allows governments and companies in industrialized countries (Annex I) to be involved in projects that can reduce emissions in developing countries. Thus, it was agreed that these countries would earn Certified Emission Reduction (CER) in order to meet the targets set in the Protocol. Each certificate is equivalent to 1MgCO_2_e and can be traded on the international financial markets [37]. These certificates are obtained by driving projects to mitigate greenhouse gases through actions promoting afforestation, reforestation and the development of technologies which promote verifiable emission reductions. Besides the CDM, the Kyoto Protocol gave rise the global carbon market, which currently constitutes one of the most important mechanisms and incentives to mitigate emissions of greenhouse gases, as it becomes the primary tool for protocol countries to meet the emission reduction targets that were agreed [19]. However, the Kyoto Protocol has had a lot of problems, specially related with its ratification. These problems and the consequences of the global economic crisis (2008–2009) produced a significant reduction in the level of industrial activity and GHG emissions in the protocol countries. Thus, the imbalance created by that falling demand and the growing supply of emissions has led the downward trend in carbon prices in the last decade [38].

There are currently two types of carbon markets: Regulated Compliance and Volunteers. The former are the result of the Kyoto Protocol (CER), and the latter are used by any country or company which wishes to undertake such projects for different reasons (reputation, corporate responsibility, etc.) but are not obligated to do so. In this case, the credits are called Verified Emission Reductions (VERs).

Nevertheless, the emission reductions associated to oceanic carbon are not yet covered under the UNFCCC; therefore they are not guaranteed under the CDM and do not result in credits that can be traded within the regulated carbon markets. Further, these reductions are not considered in the current initiatives of the voluntary market that are gaining strength in the UNFCCC, such as REDD and REDD+. Thus, the current economic incentives do not favor the protection of marine and coastal ecosystems that sequester carbon [10].

To include blue carbon in projects to reduce emissions that give rise to carbon credits, either through their inclusion in the CDM or through initiatives such as REDD+, can contribute to direct economic incentives towards the conservation of key marine and coastal ecosystems, highlighting the role these ecosystems play in the global climate and avoiding the excessive destruction thereof.

Considering the above, the price associated with one of these markets can be the tool to align the incentives mentioned, or at least provide elements for informed decision making. Thus, it can be possible to grant a monetary value to the carbon sequestration service. Theoretically, in competitive markets the price is set at the point where the buyer’s willingness to pay equals the marginal cost for the seller. In the case of carbon markets, the willingness to pay of the country that is responsible for reducing emissions is a function of the cost that can be avoided through the purchase of carbon credits [39]; generally, this cost is associated with the cost incurred in those countries to reduce emissions within their territory. Thus, the country will decide to reduce their emissions to the point where the marginal cost of reducing an additional unit of GHG starts to be higher than the price that the country has to pay to emit this additional unit of GHG [26].

As a result, under ideal conditions, the market price will reflect the value of the carbon allowances, given some supply constraints on them [27]. Therefore, carbon markets, in which these rights are traded, would grant a price that approximates these features. However, in the current carbon markets this assumption is not satisfied, because these are imperfect markets which have several distortions such as the existence of transaction costs, uncertainty about decisions on global mitigation commitments, problems in verification or monitoring projects that give rise to emission permits, among others. This causes the scenario where the price of a carbon credit is not necessarily equal to the marginal cost of polluting or emitting GHGs.

**Fig 3:**
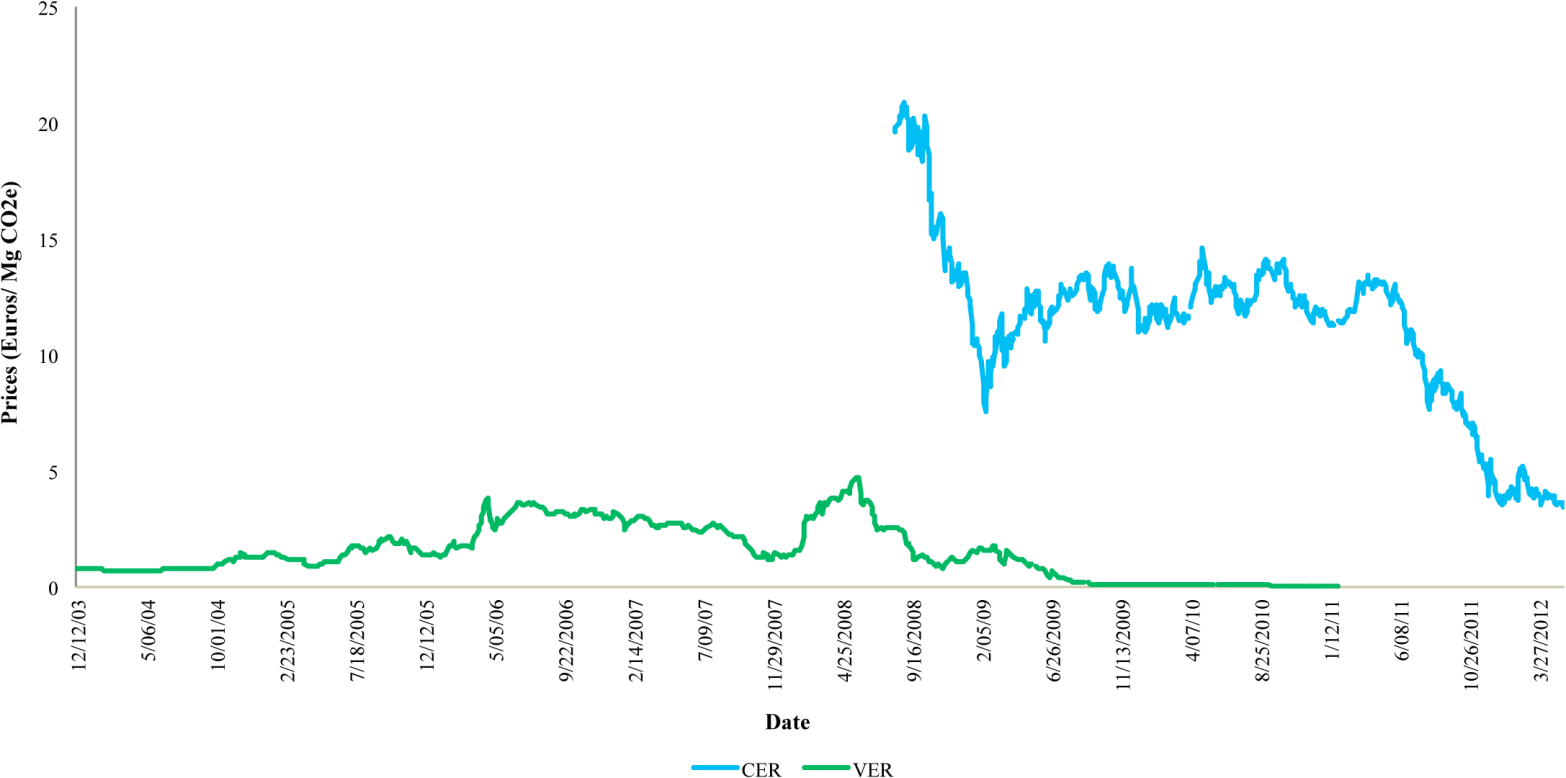
Price of Certified Emission Reductions (CER)—Phase II EU ETS (2008–2012) and Price of Verified Emissions Reductions VER (Chicago Climate Exchange (CCX) 2003–2010). **In Euros.** Source: authors’ own based on data from BlueNext [40] and ICE Closing Prices [42].

The average price of CERs that were traded in Phase II (2008–2012) of the European Union Emissions Trading Scheme (EU ETS) was €11.3/MgCO2e. However, since the end of 2011 the price of the certificates began to fall and remained near €1/MgCO2e [40] (see Fig 3). This was mainly because of four elements that have failed to generate strong incentives to invest in the CER: (i) the economic crisis that Europe experienced since the end of 2008, (ii) the uncertainty associated with the renewal of the Kyoto Protocol beyond 2012, (iii) the increase in the value of Emission Reduction Units (ERU) that are substitutes for CER credits, and (iv) mitigation targets are still so modest [41].

In contrast, the price of Verified Emissions Reductions (VER), which were traded in what was the largest voluntary market of permissions, The Chicago Climate Exchange (CCX), until 2010 was around €0.03/MgCO2e, reaching a maximum value of €4.72/Mg CO2e in 2008 [42] (see Fig 3). However, since 2011 the credits in the voluntary market began trading in markets Over The Counter (OTC), and prices associated to these loans fell significantly.

Given the limitations of the voluntary market, the price of Certified Emission Reductions can be considered as an appropriate measure of monetization of oceanic carbon fluxes, since there is currently no market for it. In addition, between the two nearest options (i.e., CER and VER) which could be better adjusted to the objectives of a government that wants to protect its marine areas, is the option framed in a regulated market. Thus, the estimated price of CER is taken as a measure to monetize carbon fluxes produced by coastal marine ecosystems.

Regarding the price series, this paper considers only the information available for the second phase of the European Emissions Market (EU ETS), mainly due to the various problems encountered in the first phase.

Now, if we consider the price at which CERs are traded as an adequate monetization measure—given that their market reference was created based on the Kyoto Protocol—it is necessary to bear in mind that this price, as it is generated on the financial market, is by nature volatile and dependent on the expectations of the agents, the success of the projects and the global economic situation, among others.

According to the Efficient-Market Hypothesis, the price of the CER and in general the price of a financial asset usually follows a random-walk behavior, meaning that the price will always be unpredictable at least in the short-term [43]. Following this theory, the historical information available with regard to the prices is appropriate to obtain a correct prediction of the future price of the asset.

To estimate the price of carbon we use the historic price series of the CER negotiated in phase II of emissions in the European market, and it is modeled as a geometric Brownian motion, which is one of the most frequently used mathematical models for modeling the process of financial assets. According to the geometric Brownian motion, the future adopting of the model would require a price simulation (1,000 times) to determine the confidence interval in which prices would be found in a given period. CER price simulations must take into account the variables associated to the carbon credit returns and their historic prices.

## Results

As discussed in the methodology section, if protection is implemented, the contribution of the subsystem to the mitigation of greenhouse gas emissions, specifically carbon dioxide, would be represented by a kind of insurance against the potential loss of carbon capture function and the potential for the release of the carbon stored in the first meter depth of the seabeds.

Following (2) and (3), and data presented in Table 2, it can be observed in Table 3 that the contribution of the new network of MPAs to the annual capture rates is between 49 and 94% additional to the contribution under the current protection scheme, which is equivalent to an approximate increase of the annual capture rates of between 6.00 thousand and 2.70 million MgCO_2_e. Insofar as storage, the additional contribution of the subsystem is between 49 and 68% additional to the current protected area, which is equivalent to keeping stored between 102.00 and 304.00 million MgCO_2_e.

**Table 3:** General contribution of the system of Marine Protected Areas to the Mitigation of GHG (MgCO_2_e/ha/yr), using Eqs (2) and (3) and data from Table 2.

Conservation status	Ecosystem	Capture rates (CR) (MgCO2e/ha/yr)			Total storage (LR) (MgCO2e/ha)		
		Min	Max	Avg	Min	Max	Avg
**Current protection**	**Mangroves**	12,212	2,252,657	1,132,434	202,121,031	439,663,641	343,296,787
	**Seagrasses**	0	625,677	312,838	6,200,913	8,337,086	5,696,415
	**Subtotal**	12,212	2,878,334	1,445,273	208,321,945	448,000,727	348,993,202
**Subsystem**	**Mangroves**	5,955	1,098,415	552,184	98,555,965	236,233,008	167,394,486
	**Seagrasses**	0	1,611,740	805,870	3,023,619	68,238,699	14,673,928
	**Subtotal**	5,955	2,710,155	1,358,055	101,579,584	304,471,707	182,068,415
**Contribution**		49%	94%		49%	68%	

Source: Authors’ own based on the annual carbon capture and storage rates per hectare (Table 2 values) and the hectares of the MPAs presented in [32]

Once the absolute contribution of the subsystem to the mitigation of greenhouse gas emissions is determined, the next step is to examine the effect of this network of MPAs on the benefits that the protection can generate for the economy. The above, undertaken within the framework of a hypothetical market that would allow Colombia to include this project in the global portfolio, giving it the right to obtain CERs, and trade them on the international emissions market, is shown in the methodology.

**Fig 4:**
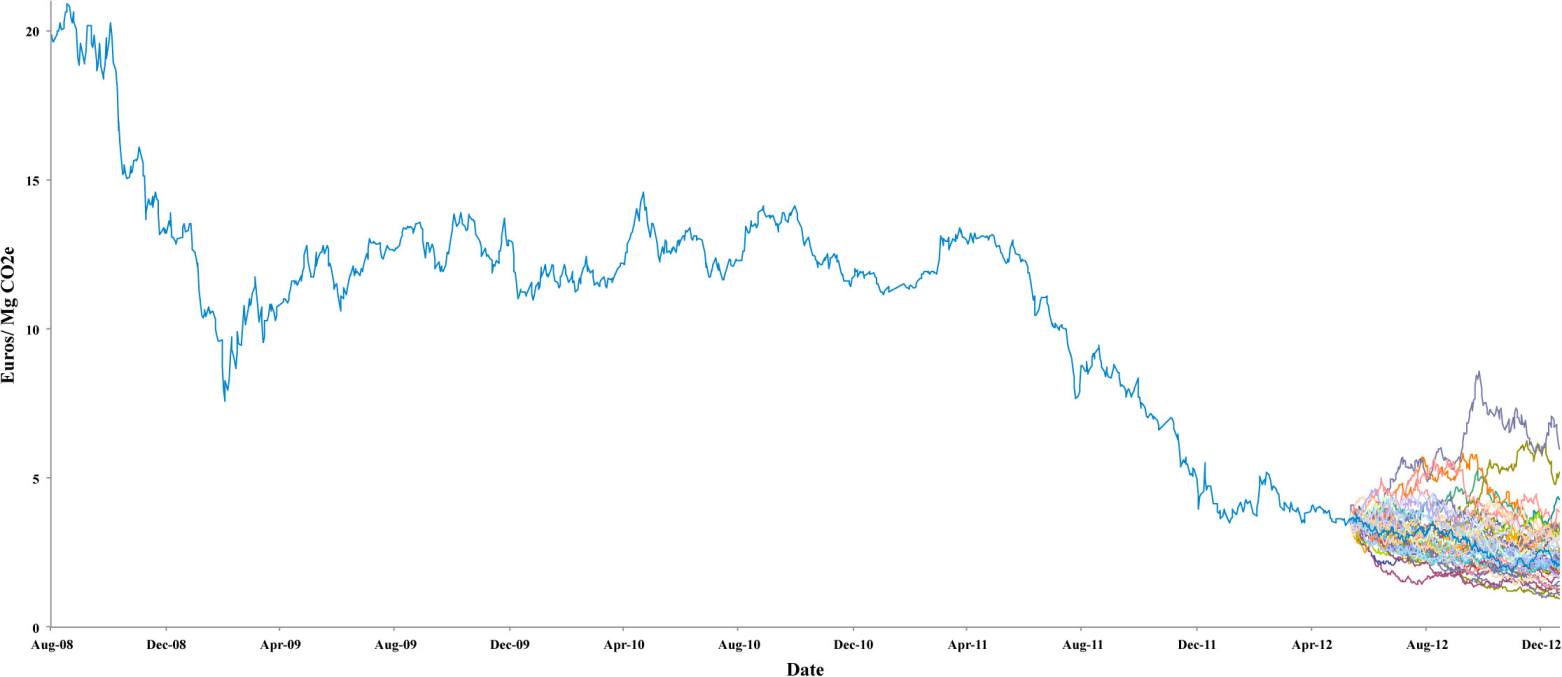
CER Price Simulation following a Brownian motion process. Source: authors’ own based on BlueNext prices data [40].

**Fig 5:**
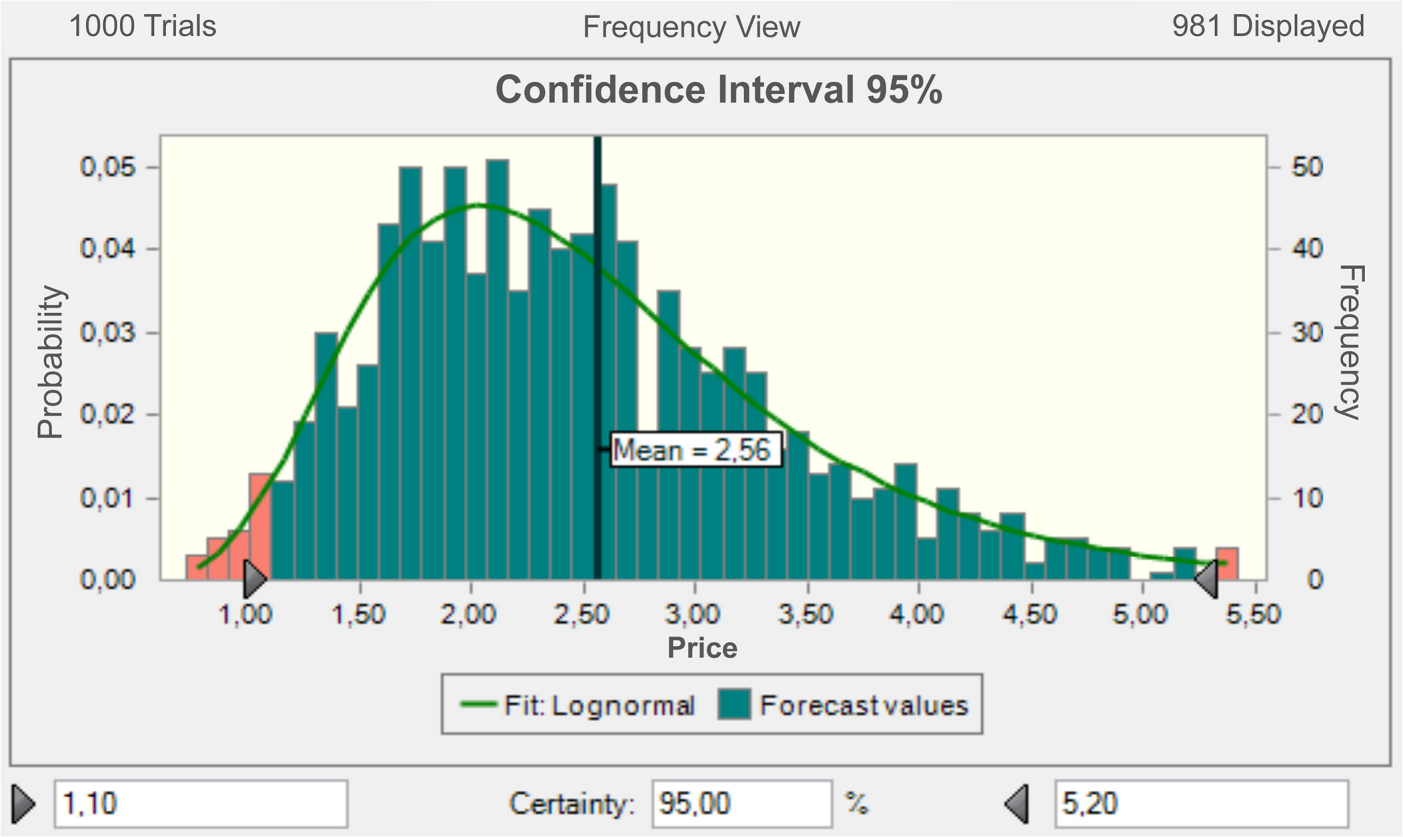
Distribution and Confidence Interval for CER Price Simulation. Source: Authors’ own based on BlueNext prices data [40].

To obtain the price estimations, we used the historic CER price series in Phase II (August 2008 to May 2012), following the proposed methodology, and we obtained a confidence interval for the price at the end of the Phase (April 12 to Dec 12). This interval is the result of 1,000 price simulations, which followed a Geometric Brownian Motion process (See Fig 4). The results obtained indicate that within a confidence level of 95%, the price of the Certified Emission Reductions in this market would be between 1.10 and 5.20 € / *tCO*_2_ *e* (See Fig 5), equivalent to 1.40 and 6.80 $ / *tCO*_2_ *e*, respectively, by the end of 2012, which coincides with the end of Phase II in this market.

With the range of prices obtained, the next step is to estimate the economic value of the services associated to the carbon sequestration. As shown in (4) the economic benefits depend on several variables such as the timespan of the project, the carbon price, the annual rates of loss of ecosystems and the discount rates.

The evaluation includes a period of eight years, beginning in 2013 and ending in 2020. The reason for this choice is based on two arguments: (i) This period coincides with the planning horizon of the Plan of Action for the establishment of the MPA’s Subsystem, which in accordance with the Colombian government’s goal, in 2019 should be fully established and articulated in the National System of Protected Areas, and (ii) the availability of data on prices of CER limits its estimate, so one can assume the validity of this estimate beyond 2020 can be a difficult and implausible argument, as it will depend on the current negotiations.

As for the price, the loss rate and the discount rate, we analyze different scenarios related to different values these variables can take. The results are presented in Table 4, where it can be seen that the associated benefits can vary between 43.77 and 294.68 million Euros depending on the values of exogenous variables. Given a price level and a discount rate, the annual loss rate does not have important effects on the benefits. As example, for a price of 1.1 € / MgCO_2_e and an annual discount rate of 4%, the gap between benefits as a result of different annual loss rates is about €345,000. As expected, discount rates have an effect in reducing the present value of benefits. But the most significant effect comes from changes in the price.

**Table 4:** Benefits in Present Value for the 2013–2020 Period Derived from Carbon Sequestration and Storage Given Different Possible Prices (P) Different Degradation Rates (x) and Different Discount Rates (Values in Euros), following Eq (4).

Discount Rates	Benefits with *x*_*i*_ = 0.58%		Benefits with *x*_*i*_ = 1.12%	
	P = 1.1	P = 5.1	P = 1.1	P = 5.1
**4%**	63,559,818	294,686,428	63,214,593	293,085,838
**6%**	54,742,698	253,807,053	54,445,363	252,428,500
**9%**	44,009,212	204,042,711	43,770,176	202,934,453

Source: Authors’ Own

In fact, as presented in the methodology, and given the relevance of the carbon price for the determination of the benefits, now we have to consider the uncertainty related to the price formation in the period of analysis. To do that, we include the term *α*, and simulate three possible values for it. The expected benefits in considering the values assigned to *α* are shown in Table 5.

**Table 5:** Annual Value of the Expected Benefits Derived from Carbon Sequestration in Different Negotiation Scenarios (Values in Euros); under two different degradation rates (x) and three different discount rates, following Eq (5).

Discount Rate	Benefits with x_i_ = 0.58%			Benefits with x_i_ = 1.12%		
	Alpha = 0.7	Alpha = 0.5	Alpha = 0.3	Alpha = 0.7	Alpha = 0.5	Alpha = 0.3
**4%**	19,739,022	26,604,769	33,470,516	19,631,810	26,460,265	33,288,721
**6%**	18,432,497	24,843,800	31,255,103	18,332,381	24,708,861	31,085,341
**9%**	16,625,523	22,408,313	28,191,104	16,535,221	22,286,603	28,037,984

Source: Authors’ own

The results indicate that the parameter *α*, associated with uncertain market elements such as negotiations to renew the Kyoto protocol, significantly affect the benefits that could be obtained within a hypothetical market thanks to the subsystem or network of MPAs. Thus, given a discount rate and an annual rate of loss, this parameter gives rise to expected benefits that may find themselves between 16 and 33.00 million Euros per year. For example, if we consider a low annual loss rate of 0.58% and a discount rate of 6%, the gap between the expected benefits of the responses to *α* = 0.7 and *α* = 0.5 (i) and (ii) exceeds 6 million per year, similar to the gap between responses associated to *α* = 0.5 and *α* = 0.3.

**Fig 6:**
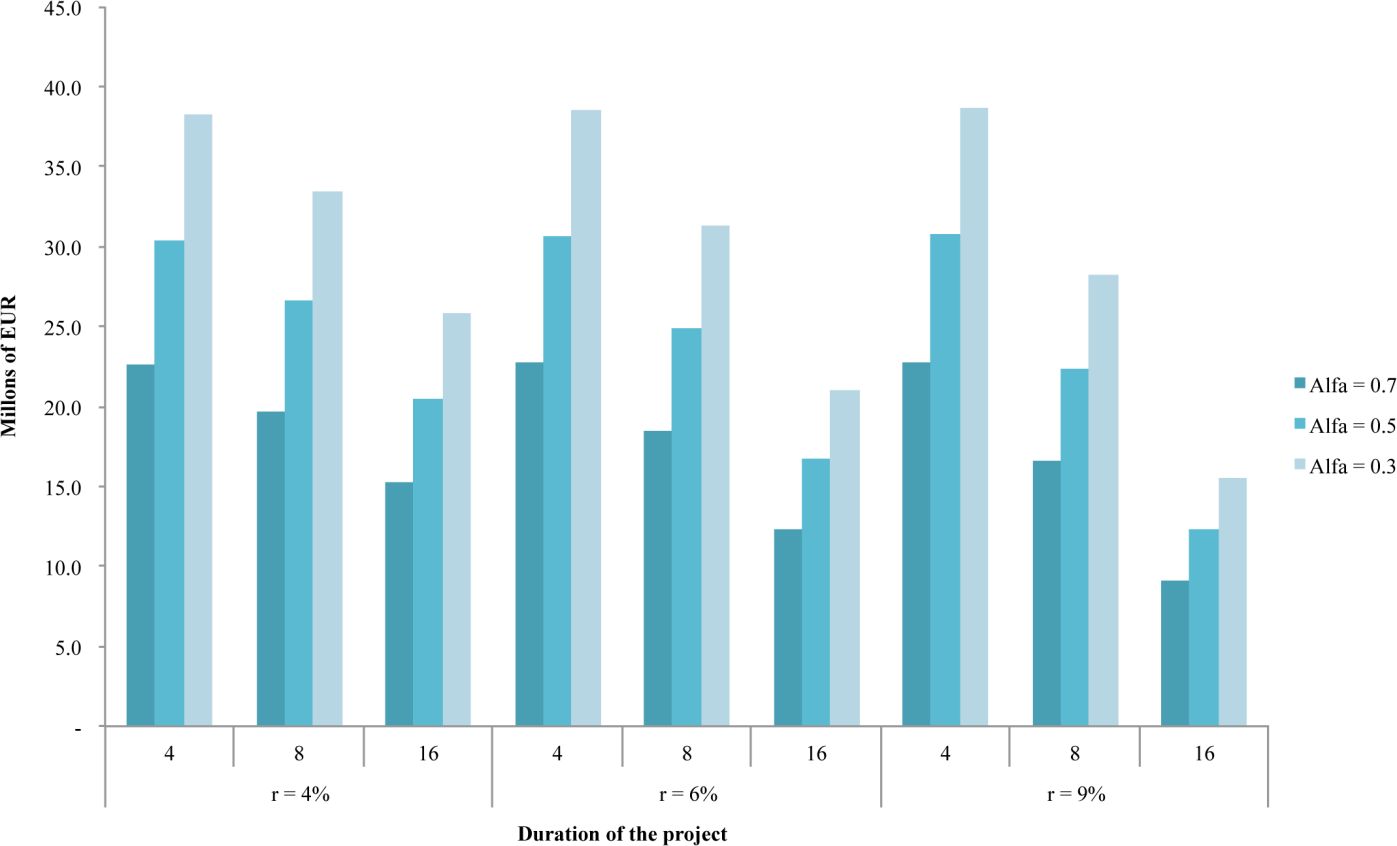
Effect of the Lifetime of a Project on the Expected Benefits.

Additionally, in Fig 6 it can be seen that benefits are dependent substantially on the length of the project: as duration increases, given a discount rate and price level, the expected benefits fall significantly. Thus, the determination of the lifetime of such projects, in the hypothetical case of presenting one of these for evaluation within the CDM, should be carefully analyzed, because otherwise they could be sacrificing profits for the country.

**Fig 7:**
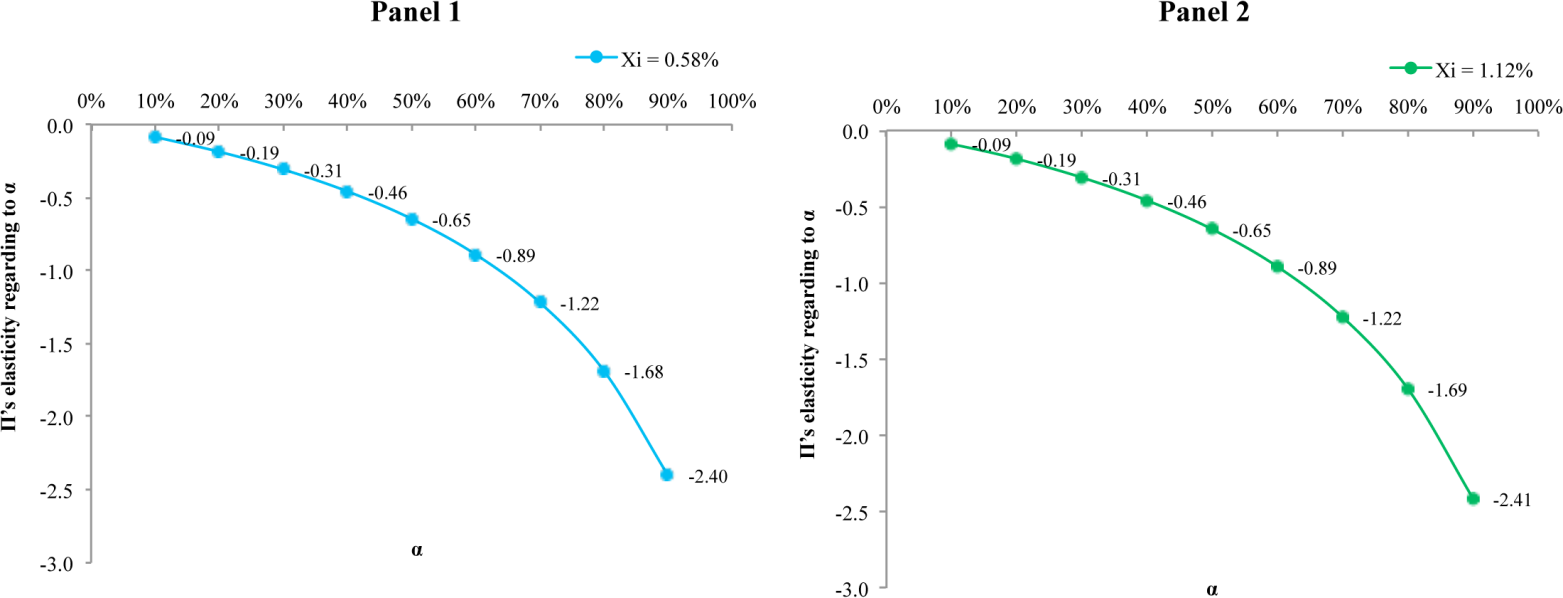
Elasticity of the benefits with respect to the parameter alpha given a discount rate equal to 6%. Panel 1 with a loss rate of 0.58%. Panel 2 with a loss rate of 1.12%.

**Fig 8:**
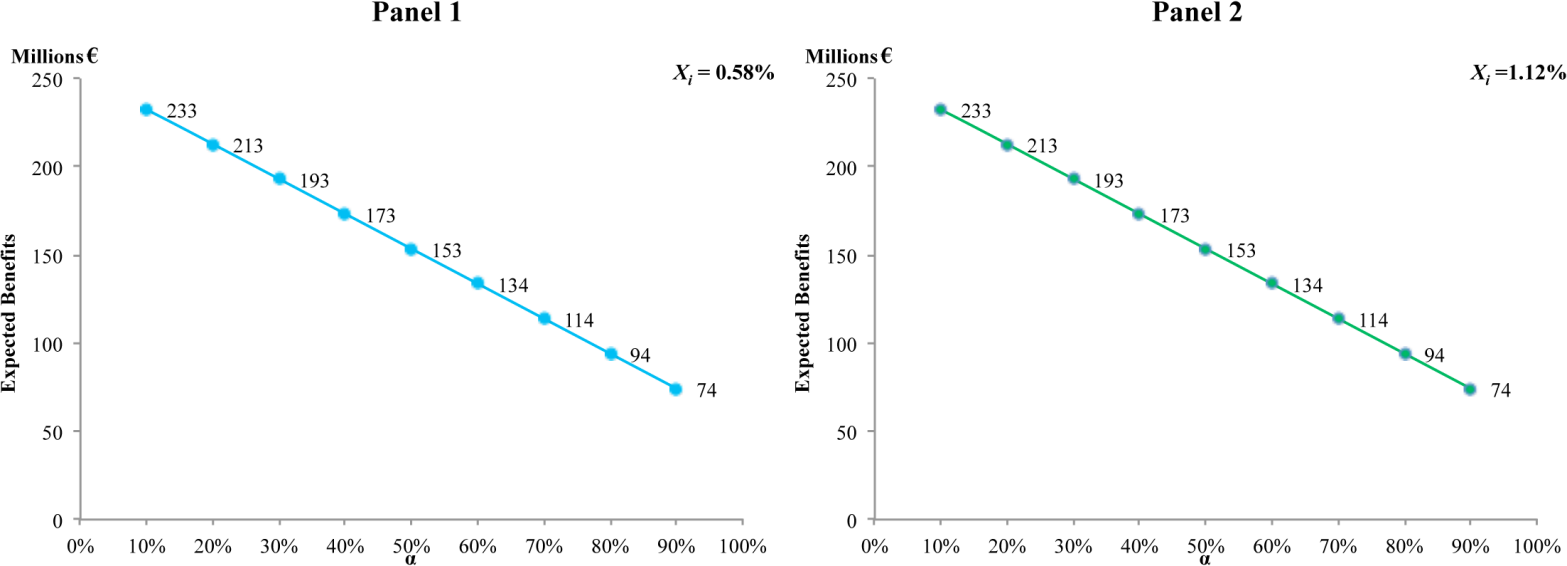
Present value of the benefits at different values of α, given a discount rate of 6%. Panel 1 with a loss rate of 0.58%. Panel 2 with a loss rate of 1.12%.

Finally, we estimate the elasticities of the expected benefits to changes in the value of *α*. Estimated elasticities can be observed in Fig 7, where it can be seen that the effect of *α* on the expected benefits, measured from the elasticity as described in (6), is initially less than proportional. However, for values of *α* greater than 0.7 this behavior changes, and with an increase of *α*, expected benefits react more than proportionally to changes in the value of the parameter. Similarly, Fig 8 shows that the present value of the expected benefits depends negatively on *α*, as expected. In both Figs (7 and 8) and panels (1 and 2) it can be observed that the loss rate *x*_*i*_ has no significant effect on these elasticities.

In contrast, the annual rate of loss does not affect significantly the benefits, as the elasticity ranges from -0.01 to -0.04 for values of annual loss from 0.5% to 2.1%, respectively. That is, even a 2 percent annual rate of loss would imply a reduction in benefits of less than 0.04%, in the worst-case scenario. Given the small effect of annual loss rate on expected benefits, the detailed results are not presented.

## Discussion

This paper has focused on presenting the methodology and the results associated with the economic valuation of the carbon sequestration service provided by the Marine Protected Areas’ subsystem for Colombia, a proposed network of MPAs that generates a bundle of ecosystem services. From the proposed methodology, the Subsystem contribution to GHG mitigation is determined, and a function of benefits which includes both economic and biological elements allowed determining the monetary value associated with this regulation service provided by marine and costal ecosystems, such as mangroves and seagrasses.

According to the estimations undertaken, the annual rates of carbon capture, considered stable for the analyzed period because they are under protection, would increase from 49 to 94% with respect to the current protection areas, while the total storage, which is also considered stable, would increase from 49 to 68%. These figures indicate that the GHG capture capacity to be retained, due to the implementation of the subsystem, would increase to values between 18 thousand and 5 million MgCO2e per year, that are equivalent approximately to 27% of the annual reductions of the MDL projects which are held in Colombia currently [44]. Likewise, the total protected marine storage, the product of the consolidated and expanded network of marine areas would reach between 309 and 928 million MgCO2e, i.e., 25% more CO2e stored than that which was issued by the Colombian industrial processes sector in 2008 [45].

In this study, possible global responses to the post-Kyoto negotiations were summarized in three alternatives: (i) some countries withdraw from the Protocol, (ii) the Protocol is ratified and (iii) the future of the Protocol is as yet undefined. Considering these options, and other aspects of the carbon market, a probability value was assigned to the realization of low or high prices of CER. Thus, given different loss rates, discount rates and estimates of future carbon price, the annual value of the expected benefits was found to be between 16 and 33 million Euros for the measurement period chosen, in this eight-year case (2013–2020). These results are encouraging for the economic incentives that could be generated in terms of the protection and conservation of marine and coastal ecosystems by environmental policy makers, because it is equivalent to 20 to 36% of the proceeds from the sale of CER Colombian projects between 2007 and 2010 [44].

Taking into account the current uncertainty about the negotiations of the Kyoto Protocol and the high volatility and low carbon prices, the results obtained are not only a tool to highlight the role that these ecosystems play in mitigating climate change, but also to show that the conservation of mangroves and seagrasses can be economically attractive.

The valuation exercise performed in this study suggests that the subsystem of marine protected areas not only protects the biodiversity, but it has positive effects in terms of strengthening strategies for adaptation and mitigation of climate change. In that sense, this work aims to put on the agendas of environmental debate the issue of marine ecosystems that historically have been overlooked.

One of the main limitations of this study is the available information on the biological characteristics of ecosystems in the country. The estimates of the carbon capture and storage were performed based on biological surveys around the world, which might not capture in detail the specific behavior of the ecosystem at these latitudes. Moreover, this research was performed while assuming the recognition of Blue Carbon within the negotiations and global carbon initiatives. Similarly, it was assumed that the expansion project of the network of marine areas could be evaluated and registered under the CDM. However, neither the former nor the latter points might be completely true. In that sense, this approach should be seen as a tool for composing the valuation elements rather than a completely real scenario of rents generation for the country.

In addition, problems in recent years have had to face the Kyoto Protocol, due to the international financial crisis (2008–2009) and the inability of the commitments of signatory countries to reduce emissions effectively, have generated distrust in this market. Therefore, the results obtained through the proposed valuation exercise in this document may differ somewhat when considering these elements. The current negotiations taking place in the Conferences of the Parties (COP) will help to clarify the future of these markets for the next decade.

In contrast, the inclusion of the total area of mangroves and seagrasses of the Subsystem in the assessment of oceanic carbon could be overestimating the benefits provided by these ecosystems, as currently serving regardless of the existing protection figure. That is, as a reviewer pointed out, additionality might be questioned. However, the framework under which the services are in these ecosystems in the proposed methodology assumes that in order to enter into the hypothetical trading in the carbon market, there must be some emissions reduction project, in this case the protection figure represented by the subsystem. If the areas currently serving capture and storage were not protected, they may not be classified as accepted projects within market mechanisms, therefore it would not be possible to obtain expected profits arising under the methodology proposed.

Additionally, when we take the annual rate of loss of ecosystems as a constant we are ignoring the temporal dynamics of these ecosystems, such as growth rates and carrying capacity, among others. However, this assumption was necessary due to the lack of detailed information regarding specific features of these ecosystems and their behavior over time.

Active negotiations carried out in the international scope to include REDD and REDD+ initiatives within the portfolio of regulated projects should be followed carefully. The possible approval of such initiatives leads to credits competition with CER, and thereby could affect the dynamics of prices in the regulated market, as has happened with the increased supply of the ERUs, forcing probably to adjust the valuation exercise performed in this study, but also opening up new possibilities for the oceanic carbon market.

Another aspect to consider is that despite the weakness of the international carbon markets, both developed and developing countries are integrating initiatives to carbon price fixing. At present, there are several regional trade schemes and carbon taxes that are underway, while new pricing mechanisms are under development [46]. Some countries are hosting pilot projects for the development of new market mechanisms, such as the Regional GHG Initiative (RGGI) Cap-and-Trade System of Quebec, and the Western Climate Initiative of California [46]. Likewise, the World Bank continues to strengthen its post-2012 initiatives in looking to secure additional resources and incentives to the Kyoto Protocol in order to provide technical and financial support and help countries to explore and implement elements favoring GHG mitigation, including setting mechanisms domestic prices, new instruments for carbon credits and carbon taxes. Among these initiatives are: the Forest Carbon Partnership (FCP), the Carbon Partnership Facility (CPF), the Partnership for Markets Readiness (PMR) and the Bio-Carbon Fund [38].

Finally, it is useful to recall that carbon sequestration is not the only value associated to mangroves and seagrasses. There are many ecosystem services that these ecosystems provide and that generate value for to society, values that are not included in this study. There are recent studies estimating other values of Colombian marine and coastal ecosystems being protected [47 48 49 50 51 52]. Similarly, analysis of the costs of protection of the new network is not considered in this study.
